# Economic Inequalities in Maternal Health Care: Prenatal Care and Skilled Birth Attendance in India, 1992–2006

**DOI:** 10.1371/journal.pone.0013593

**Published:** 2010-10-27

**Authors:** Praveen Kumar Pathak, Abhishek Singh, S. V. Subramanian

**Affiliations:** 1 International Institute for Population Sciences, Mumbai, Maharashtra, India; 2 Department of Public Health and Mortality Studies, International Institute for Population Sciences, Mumbai, Maharashtra, India; 3 Department of Society, Human Development and Health, Harvard School of Public Health, Boston, Massachusetts, United States of America; Kenya Medical Research Institute, Kenya

## Abstract

**Background:**

The use of maternal health care is limited in India despite several programmatic efforts for its improvement since the late 1980's. The use of maternal health care is typically patterned on socioeconomic and cultural contours. However, there is no clear perspective about how socioeconomic differences over time have contributed towards the use of maternal health care in India.

**Methodology/Principal Findings:**

Using data from three rounds of National Family Health Survey (NFHS) conducted during 1992–2006, we analyse the trends and patterns in utilization of prenatal care (PNC) in first trimester with four or more antenatal care visits and skilled birth attendance (SBA) among poor and nonpoor mothers, disaggregated by area of residence in India and three contrasting provinces, namely, Uttar Pradesh, Maharashtra and Tamil Nadu. In addition, we investigate the relative contribution of public and private health facilities in meeting the demand for SBA, especially among poor mothers. We also examine the role of salient socioeconomic, demographic and cultural factors in influencing aforementioned outcomes. Bivariate analyses, concentration curve and concentration index, logistic regression and multinomial logistic regression models are used to understand the trends, patterns and predictors of the two outcome variables. Results indicate sluggish progress in utilization of PNC and SBA in India and selected provinces during 1992–2006. Enormous inequalities in utilization of PNC and SBA were observed largely to the disadvantage of the poor. Multivariate analysis suggests growing inequalities in utilization of the two outcomes across different economic groups.

**Conclusions:**

The use of PNC and SBA remains disproportionately lower among poor mothers in India irrespective of area of residence and province. Despite several governmental efforts to increase access and coverage of delivery services to poor, it is clear that the poor (a) do not use SBA and (b) even if they had SBA, they were more likely to use the private providers.

## Introduction

Maternal mortality, a crisis essentially of the poor in 21^st^ century [1], and a neglected tragedy of developing countries [2], reflects one of the shameful failures of human development [3]. The gap in the risk of maternal deaths between developed and developing countries is considered the “greatest health divide in the world” [4]. The emphasis on two out of eight critical United Nations Millennium Development Goals, that is, reducing *under five mortality* by two-thirds between 1990 and 2015; and reducing *maternal mortality ratio* by three quarters between 1990 and 2015 epitomise the relevance of these indicators in global efforts towards human development and alleviation of poverty [5], [6], [7]. It also underlines the important linkage between improvement in maternal health and the development process, as poor maternal health may affect child health negatively, reduce women's productive capacity, lower participation in economic activities, and sabotage the poverty alleviation programme [8]. However, monitoring the progress towards reduction in maternal mortality particularly in developing countries is difficult due to paucity of reliable health information and incomplete vital registration systems [9]. This had led to the use of alternative process indicators, like proportion of skilled birth attendance, for monitoring progress [10], [11].

Recent global estimates of maternal mortality indicate that more than half a million women died due to pregnancy related causes in 2005 [12]. Approximately 80% of the maternal deaths globally occur due to haemorrhage, sepsis, unsafe induced abortion, hypertensive disorder of pregnancy, and obstructed labour [13]; these deaths are unjust and can be avoided with key health interventions, like provision of antenatal care and medically assisted delivery [14], [15]. In addition, the risk of maternal death was not uniformly distributed, as the large proportions of these maternal deaths are concentrated in developing countries. Of the total maternal deaths in 2005, 99% occurred in the developing world, and Sub-Saharan Africa and South Asia alone accounted for 86% of the total global maternal deaths [12]. Despite declining maternal mortality owing to large-scale programmatic interventions over the past two decades, the progress has been slow and uneven, both across and within countries. [16], [17], [18], [19], [20], [21], [22], [23]. Conspicuous variations in maternal mortality are reflected through inequities in access to maternal health care such as prenatal care, skilled birth attendance, and post natal care on various economic, geographic and social scales [24], [25], [26], [27], [28], [29], [30], [31], [32], [33], [34].

India continues to have unacceptably high levels of maternal mortality despite its remarkable economic growth and impressive advancement in the fields of science, agriculture, medicine and information technology. The maternal mortality ratio in India was 16 times higher than that of Russia, 10 times that of China and 4 times higher than that of Brazil in 2005 [35]. Among developing countries, India contributes the largest number of births per year (27 million) in the world and accounts for 20% of global maternal deaths [36]. This magnitude clearly suggests that India's progress towards reducing maternal mortality will be crucial in the global achievement of Millennium Development (MDG-5). But inadequate maternal health care services with poor organization, huge rural-urban divide, large interstate disparities coupled with stringent social-economic and cultural constraints demands a significant shift in programme priorities to increase service coverage and accessibility to all sections of population [37], [38], [39], [40], [41], [42], [43], [44], [45], [46].

**Figure 1:**
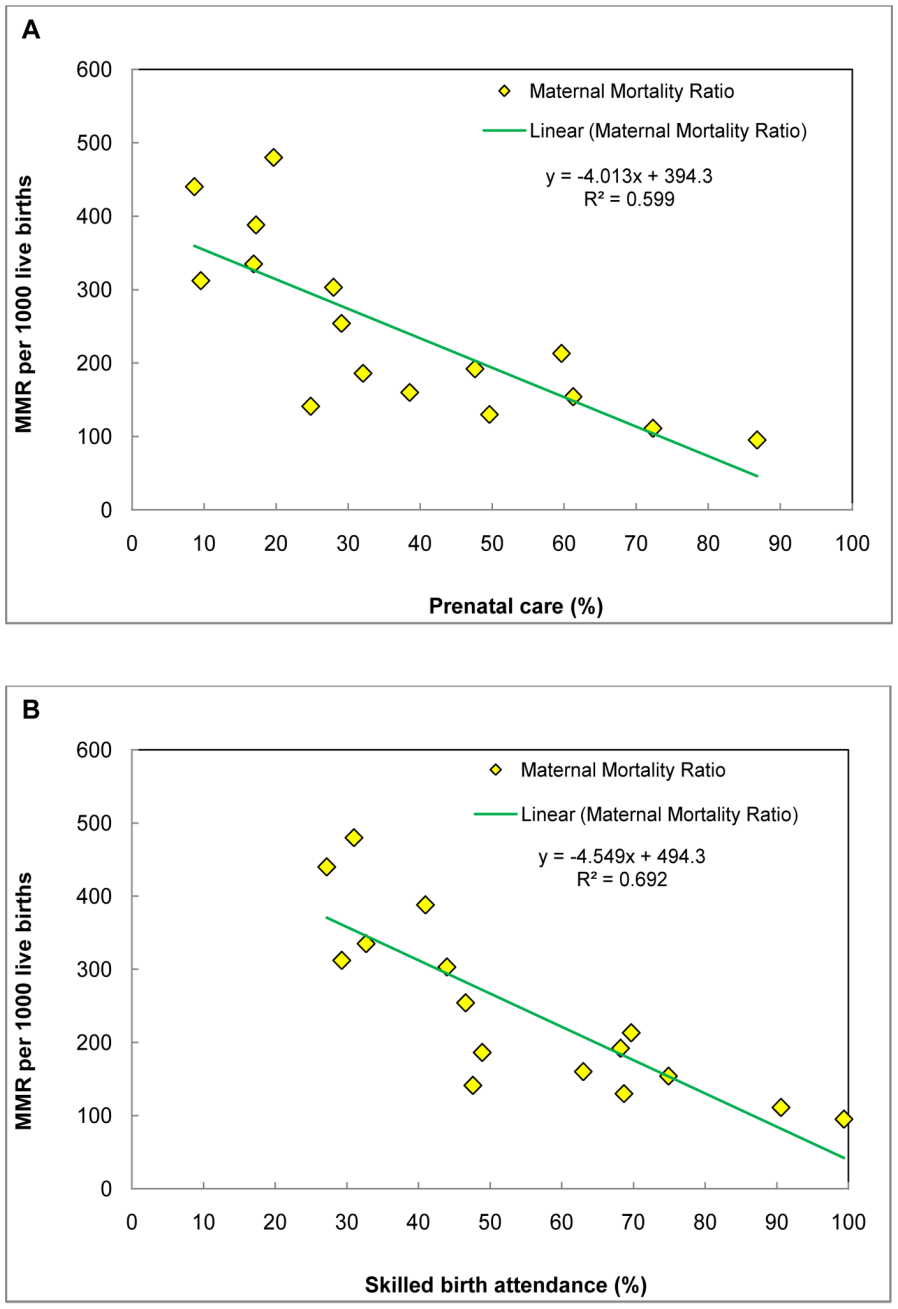
Association between maternal health care (prenatal care and skilled birth attendance) and maternal mortality ratio across 15 major states, India. **A**. X axis = Prenatal care (%). Y axis = Maternal mortality ratio (MMR) per 1000 live births. **B**. X axis = Skilled birth attendance (%). Y axis = Maternal mortality ratio (MMR) per 1000 live births.

According to recent estimates, nearly 28 percent of the Indian population lived below the poverty line with large inter-state variations [47]. Poverty is largely concentrated in the central and eastern states of India, namely, Uttar Pradesh, Madhya Pradesh, Chhattisgarh, Bihar, Jharkhand and Orissa where poverty is significantly higher than the national average, and these states together account for 55% of the total poor population in India. Importantly, most of these states also contribute to nearly half of the maternal deaths in India during 2004–06 [48], [49]. The use of maternal care services is relatively limited in these states [50]. On the contrary, most of the western and southern states of India, namely, Maharashtra, Gujarat, Andhra, Karnataka, Tamil Nadu and Kerala are economically and demographically advanced than the northern and eastern states [51], [52], [53], [54], [55].These states accounted for a miniscule 17% of maternal deaths with a relatively higher use of maternal care services in the country in 2004–06 [48], [50]. Studies have documented a negative association between the use of maternal care and maternal mortality ratio [56], [57]. This relationship holds true in the case of India as well, if we draw a scatter plot taking maternal health care [50] on the x-axis and maternal mortality ratio [48] on the y-axis (**Figure 1**). This highlights that any periodic scrutiny of inter-state differentials in the use of maternal health care disaggregated by area of residence remains critical for the monitoring the improvement of maternal health scenario in India.

Several scientific research and intervention studies in the past few decades have identified three key elements to reduce maternal mortality and improve neonatal health, that is, *family planning*, *skilled birth attendance for all deliveries*, and *access to emergency obstetric care for all women with life threatening complications* [58]. Interestingly, India was the first country in the world to launch the official Family Planning Programme (FPP) in 1951 with a focus on checking population growth. This FPP was later renamed Family Welfare Programme (FWP) in 1977 integrating the maternal and child health component, realising the positive association between falling birth rate and improved infant and child survival. India further revamped the Maternal and Child Health (MCH) programme to Child Survival and Safe Motherhood Programme (CSSM) in 1992 in tune with the “International Safe Motherhood Conference-global undertaking to reduce maternal mortality”, held in Kenya in 1987. The CSSM programme was later integrated into the Reproductive and Child Health (RCH) programme in 1997–98 to improve the maternal and child health and to meet the needs of family planning services, especially among the poor and the underprivileged. In the same league, the recently launched National Rural Health Mission (NRHM, 2005–12), a flagship programme of the central government of India, focuses on providing effective health care to the rural population throughout the country, with special emphasis on eighteen states with weak public health infrastructure. The NRHM attempts to synergise health issues with determinants of health, like sanitation and hygiene, nutrition and safe drinking water. Most critically, it aims at improving the availability, accessibility, affordability, and quality of effective health care services to rural population, particularly among poor and underserved women and children. It also envisages reducing the large economic and inter-state disparities in the access to public health care, underlining the lead role of the public sector in revamping public health infrastructure, integrating the local traditional system of medicine i.e., Ayurveda, Yoga and Naturopathy, Unani, Siddha and Homeopathy (AYUSH) and regulating the private health sector.

Previous studies have highlighted the socioeconomic gradient in the utilization of maternal health care in the context of developed and developing countries. However, few studies have been carried out to understand the trends and regional patterns of socioeconomic differentials in the utilization of maternal health care services in India from the equity perspective. It is important to understand trends and regional dimensions of socioeconomic inequalities in maternal health care across rural-urban sub-groups of population in order to monitor policy indicators and targeted intervention programmes. Therefore, the present study is an endeavour to investigate the economic inequalities in the utilization of prenatal care and skilled birth attendance in India, and three contrasting states, namely, Uttar Pradesh, Maharashtra and Tamil Nadu by residence (urban vs. rural) during 1992–2006. An attempt has been made to quantify the relative contribution of public and private health care providers in safe-motherhood programme. Finally, the role of salient socioeconomic, demographic and cultural factors has been examined to understand the inequalities in the utilization of prenatal care and skilled birth attendance.

The three culturally and socioeconomically contrasting states of Uttar Pradesh, Maharashtra and Tamil Nadu were included to highlight the regional dimensions of socioeconomic inequalities within country and between states, considering that all the three states significantly vary in their socioeconomic, demographic, geographic and cultural profiles [50]. Uttar Pradesh is the most populous state of India situated in the central part of the country, presently passing through the early stage of demographic transition, with an estimated death rate of 30 per 1000 population and an infant mortality rate (IMR) of 71 per 1000 live births [59]. A large proportion of the state's population suffers from poverty, with low female literacy and low women autonomy. In the Human Development Index (HDI), it ranked 14^th^ among the 15 major states in India [60]. On the other hand, Maharashtra situated in the western part of India, is the second most populous states with relatively higher socioeconomic development, as it ranked fourth among 15 major states in HDI. It has almost reached the replacement level fertility with a birth rate of 18 per 1000 population and IMR of 35 per 1000 live births [59]. Tamil Nadu is among the most advanced Indian states in terms of socioeconomic and demographic parameters. It has already achieved replacement level fertility, along with low infant and child mortality and high use of reproductive and child health services. It ranked third among 15 major states in HDI in India [60] (also see **Table 1**).

**Table 1:** Socioeconomic and demographic profile of the population of India and three selected states of Uttar Pradesh, Maharashtra, Tamil Nadu.

Indicators	India	Uttar Pradesh	Maharashtra	Tamil Nadu
Population (in millions)^a^	1028.6	166.2	96.0	62.0
Density of population (people/km^2^)^a^	324	689	315	480
Urban population (%)^a^	27.8	20.8	42.4	44.0
Sex Ratio^a^ (females/1000 males)	933	898	922	987
Decadal Growth^a^ (%)	21.5	25.9	22.7	11.7
Crude Birth Rate^b^ (births/1000 mid-year population)	23.1	29.5	18.1	15.8
Crude Death Rate^b^ (deaths/1000 mid-year population)	7.4	8.5	6.6	7.2
Life expectancy at birth, male (in years)^d^	62.6	60.3	66.0	65.0
Life expectancy at birth, female (in years)^d^	64.2	59.5	68.4	67.4
Total Fertility Rate^c^	2.7	3.9	2.1	1.6
Infant Mortality Rate^b^ (infant deaths/1000 live births)	55	69	34	35
Maternal Mortality Ratio^g^ (maternal deaths/100,000 live births)	254	440	130	111
Female Literacy Rate^a^ (%)	53.7	42.2	67.0	64.3
Per capita income (INR)^e^	29524	14663	41331	35134
State Human Development Index^h^	-	14	4	3
Population below poverty line^f^ (%)	27.5	32.8	30.7	22.5

a ORGI, 2004;

b Sample Registration System Bulletin (SRS), Vol 43, No.1, October 2008, Registrar General, Government of India, New Delhi;

c Sample Registration System (SRS), Statistical Report 2007, Office of the Registrar General, Government of India, New Delhi;

d SRS Abridged Life Table 2002–06, Office Registrar General of India, Ministry of Home Affairs, New Delhi;

e Economic Survey, 2008–09, Ministry of Finance, Economic Division, Government of India, New Delhi;

f INR- Indian national rupee, estimates of the National Sample Survey Organization (NSSO), 2004–05;

g MMR- Special Bulletin on Maternal Mortality in India-2004–06, SRS, Office of Registrar General, India, Vital Statistics Division, New Delhi;

h National Human Development Report (2002), Planning Commission, Government of India. Yojana Bhavan, Sansad Marg, New Delhi.

## Methods

### Data

The data for the present study is taken from the three rounds of National Family Health Survey (NFHS) conducted during 1992–93, 1998–99 and 2005–06 [50], [61], [62] (IIPS & Macro International, 2007; 2000; 1995). These surveys are nationally representative and cover more than 99% of the Indian population. These surveys are the Indian version of the Demographic Health Survey (DHS), and provide consistent and reliable estimates of fertility, mortality, family planning, utilization of maternal and child health care services, and other related indicators at both the national and state levels.

The survey adopted a two-stage sample design in most rural areas and a three-stage sample design in most urban areas. In rural areas, the villages were selected at the first stage by using the Probability Proportional to Size (PPS) sampling scheme. The required number of households was selected at the second stage using systematic sampling. In urban areas, blocks were selected at the first stage, census enumeration blocks (CEB) containing approximately 150–200 households were selected at the second stage, and the required number of households were selected at the third stage using systematic sampling technique (For details regarding sampling, see IIPS & ORCMacro 2007). A similar sampling scheme was adopted in all the three rounds of NFHS. More than 90,000 households were interviewed in each round of the NFHS. So, the different rounds of NFHS provide sufficiently large sample sizes to carry out the analysis at the national and state levels. Even the sample sizes were fixed in such a way that estimates could be provided at both the national and state levels.

The data were collected using different interview schedules, including household schedule, eligible women schedule, and village schedule in NFHS I and II. In NFHS III, men schedule was also canvassed along with the above three schedules. The interview schedules were almost similar in the three rounds of NFHS with some additions or deletions. The household response rate in NFHS III was 96 percent or higher in all the states. The individual response rate was 95 percent for the country as a whole. The response rate for eligible women varied from 90 percent in Maharashtra and Meghalaya to 99 percent in Madhya Pradesh and Chhattisgarh. The household and eligible women responses rates in NFHS I were 96 percent respectively. The eligible women response rates varied from 92 percent in Tripura to above 97 percent in Kerala [62]. The response rate in NFHS II was above 90 percent and similar to the response rates observed in NFHS I and III.

The NFHS used a multistage sampling design-the design being self-weighting only at the domain level; the domains being urban and rural areas of each state, and slum and non-slum areas of eight selected cities in NFHS III, and urban and rural areas in NFHS I and NFHS II. Therefore, it is important to use appropriate weights to make the estimates representative and comparable over the two survey rounds. We, therefore, use appropriate weights already given in the three rounds of NFHS while generating all the estimates presented in the paper [50], [61], [62]. The details of the sampling weights are given in the NFHS reports of various rounds.

### Outcome variables

The present study measures two outcomes variables, namely, prenatal care in the first trimester with four or more antenatal care (ANC) visits, in line with the gold standard definition recommended by the World Health Organization [63], and skilled birth attendance. The NFHS 2005–2006 collected information regarding ANC visits for the last birth in the five years preceding the survey; the NFHS 1998–1999 collected information for the last two births in the three years preceding the survey; and the NFHS 1992–1993 collected information for the last three births in the four years preceding the survey. To make the estimates comparable, prenatal care visits in the first trimester and four or more ANC visits for only the last live birth during the three years preceding the survey period were analyzed.

During 1998–1999 and 2005–2006 survey rounds, the questions on births attended by skilled health professionals were put to mothers regarding the last two and last three births with a reference period of three and five years respectively, while in the 1992–1993 survey, it was put to mothers for three births during the last four years preceding the survey. To make the estimates comparable, the births attended by skilled health professionals for the last two births in the three years preceding the survey were estimated uniformly for all three NFHS rounds. Births assisted by medical professionals, such as a doctor, an Auxiliary Nurse Midwives (ANM)/nurse/midwife/Lady Health Visitor (LHV) or other health personnel, and institutional deliveries are termed ‘skilled birth attendance’. The analytical sample size used in the study is given in **Appendix S1**.

### Socioeconomic and demographic predictors of prenatal care and skilled birth attendance

The present study includes a list of theoretically pertinent socioeconomic and demographic predictors in the analyses, such as the economic status of mother (poor vs., non poor), maternal education (no education, primary, secondary, higher), age of mother at delivery (in completed years, coded as <20y, 20–29y, > = 30y), paternal education (no education, primary, secondary, higher), parity(1, 2–3, > = 4), pregnancy complications (no vs. any pregnancy complication), mass media exposure (no vs. any exposure), caste groups (scheduled caste/scheduled tribe, non-scheduled caste/non-scheduled tribe), religion (Hindu, Muslim, Others), state (all 29 Indian states), and time dummies (1992–1993, 1998–1999, 2005–2006. Based on the bargaining literature on household decisions, the age difference between woman and household head (coded as <20y, > = 20y) is used uniformly across three NFHS rounds as proxy to measure the status of woman [64], since the standard variables for capturing the women's autonomy/decision making power were not uniformly available, particularly in the first round of NFHS. Earlier studies have also used ‘age difference between husband and wife’ as an indicator of women's autonomy [65], [66], [67], [68] citing the fact that women's autonomy is likely to be lower when the age-gap is higher and vice-versa. This particularly holds true in the Indian society as well, where women enjoy more agency in the household as they tend to age and undergo transition from being daughter-in-law to mother-in-law. Furthermore, research on women's autonomy in South Asia suggests that when women have greater autonomy, they are more likely to use maternal health care [69], [70].

We have constructed the wealth index for India and the states of Uttar Pradesh, Maharashtra and Tamil Nadu, separately for urban and rural areas, for all three rounds of NFHS. The wealth status is estimated from a set of economic proxies [71], [72], [73], [74], [75], by using the Principal Component Analysis (PCA). We used a similar set of durable asset ownership, access to utilities and infrastructure, and housing characteristics variables for all three rounds of NFHS. From the composite wealth index, a percentile distribution of wealth score was estimated, and the cut-off point for the poor and non-poor were generated for rural and urban areas separately, using country and state-specific poverty estimates [47]. The analysis was carried out using Stata 10 [76].

### Statistical Analysis

The entire analysis was carried out for rural, urban and combined sample of births using appropriate sampling weights. We estimated the weighted prevalence of prenatal care and skilled birth attendance by economic status of mothers and place of residence using national weights during 1992–2006. We estimated the concentration curve (CC) and concentration index (CI) to depict the inequalities in utilization of maternal health care by economic status [72]. A concentration index is a measure of socioeconomic inequality and is defined as twice the area between the concentration curve and the diagonal, and it varies between −1 to +1. The closer the value to 1 (absolute), the more unequal is the maternal health care (prenatal care and skilled birth attendance) and the closer the value to 0, more equal is the distribution of maternal health care.

Owing to the comparable sampling designs [77], [78] of the three rounds of NFHS, we have pooled the datasets to examine the effect of time dummies on the likelihood of using maternal health care. We fit the binary regression model to assess adjusted effects of socioeconomic, demographic and cultural characteristics on the likelihood of using prenatal care (used prenatal care = 1; otherwise = 0). Skilled birth attendance is then analysed in two steps. First we run a binary logistic regression model to understand the effects of socioeconomic, demographic and cultural variables on the likelihood of use of skilled birth attendance (used skilled birth attendance = 1; otherwise = 0). In the second stage, we run a multinomial logistic regression model to understand the effects of socioeconomic, demographic and cultural predictors on the likelihood of choosing the context of skilled birth attendance (home delivery assisted by skilled health professionals = 1; delivery at public facility = 2; delivery at private facility = 3). We also generated an interaction term between economic status of mothers and historical time periods to understand the changes in likelihood of seeking maternal health care by poor and nonpoor mothers over the three survey rounds. We present the results of logistic regression and multinomial regression models as predicted probabilities to avoid the complexity in interpretations of interaction term in the regression models.

### Ethical Review

The National Family Health Survey was conducted under the scientific and administrative supervision of the International Institute for Population Sciences, (IIPS) Mumbai, India. The IIPS is a regional center for teaching, training and research in population studies, and is associated with the Ministry of Health and Family Welfare, Government of India. The institute conducted an independent ethics review of NFHS protocol. Data collection procedures were also approved by the ORC Macro institutional review board. The study was reviewed by Harvard School of Public Health Institutional Review Board and was considered as exempt from full review as the study was based on an anonymous public use data set with no identifiable information on the survey participants.

## Results

### Trends, Differentials and Economic Inequalities in Prenatal Care (PNC)

Findings suggest that the utilization of prenatal care (PNC) among mothers in India, on average, increased by 12 percentage points during 1992–2006 (from 17.4% in 1992–1993 to 29.1% in 2005–2006) (**Table 2**). This increase was mainly observed due to relatively large improvement in the use of PNC among non-poor mothers (from 23.5% in 1992–1993 to 35.3% in 2005–2006) than their poor counterparts (from 6.1% in 1992–1993 to 6.2% in 2005–2006). The use of PNC also varied significantly across states in India during the study period. On average, the use of PNC ranged from the highest in Tamil Nadu (39%, 52% & 72% in 1992–1993, 1998–1999 & 2005–2006 respectively) to the lowest in case of Uttar Pradesh(6%, 5% & 9% in 1992–1993, 1998–1999 & 2005–2006 respectively). However, across all the study states, the use of PNC remained substantially lower among poor mothers than their non-poor counterparts. For example, the use of PNC among poor mothers increased by 27 percentage points in Tamil Nadu (from 22% to 49% during 1992–2006), 12 percentage points in Maharashtra (from 10% to 22% during 1992–2006) & remained unchanged in Uttar Pradesh during 1992–2006. On the other hand, among nonpoor mothers, the use of PNC increased by 30 percentage points in Maharashtra (from 28% to 58% during 1992–2006), 29 percentage points in Tamil Nadu (from 48% to 77% during 1992–2006), and only 3 percentage points in Uttar Pradesh (from 8% to 11% during 1992–2006) during the study period.

**Table 2:** Trends in prenatal care and skilled birth attendance (natal care) among poor and non-poor mothers across selected states, India, 1992–2006.

Indicators	Rural			Urban			Total		
Prenatal care (% PNC)	Poor	Non-poor	Total	Poor	Non-poor	Total	Poor	Non-poor	Total
**India**									
1992–93	6.2	18.2	12.7	7.6	35.2	33.1	6.1	23.5	17.4
1998–99	5.3	21.2	15.8	5.9	45.3	44.0	5.3	28.0	22.0
2005–06	6.1	26.8	21.1	9.8	53.2	52.0	6.2	35.3	29.1
**Uttar Pradesh**									
1992–93	1.6	4.7	3.2	3.9	17.8	16.9	1.7	8.1	5.5
1998–99	0.7	4.1	2.8	0.0	19.2	18.3	0.7	7.5	5.3
2005–06	1.9	7.3	5.6	5.4	21.6	21.1	2.0	11.1	8.7
**Maharashtra**									
1992–93	10.9	23.2	16.3	6.3	30.4	28.8	9.9	27.5	21.2
1998–99	5.3	30.3	21.0	22.4	44.6	44.1	6.3	38.0	30.0
2005–06	21.1	49.7	39.2	6.4	63.3	60.9	21.5	57.9	49.5
**Tamil Nadu**									
1992–93	21.5	43.0	33.1	20.7	52.1	49.7	21.7	48.2	39.1
1998–99	32.8	49.3	44.8	22.5	71.1	67.0	32.5	57.8	52.4
2005–06	50.5	72.2	67.1	33.4	82.5	78.6	48.6	77.0	72.3

Safe delivery (% SBA)	Rural			Urban			Total		
**India**									
1992–93	16.0	34.7	26.8	36.1	70.1	67.6	17.1	46.4	36.2
1998–99	16.5	43.1	34.1	36.8	74.9	73.8	16.9	52.1	42.9
2005–06	18.4	48.5	40.2	31.4	77.8	76.5	18.9	57.8	49.5
**Uttar Pradesh**									
1992–93	8.0	16.5	12.4	28.1	45.8	44.8	8.4	24.2	17.8
1998–99	8.6	22.5	17.0	24.2	53.6	52.3	8.8	29.3	22.7
2005–06	13.5	27.5	23.1	15.6	52.2	51.2	13.6	34.1	28.8
**Maharashtra**									
1992–93	27.5	53.3	39.0	43.6	83.3	80.7	28.0	70.3	55.2
1998–99	21.8	57.1	43.9	30.3	85.6	84.6	23.7	71.8	59.7
2005–06	39.7	69.4	58.2	35.5	90.8	88.4	40.2	82.2	72.2
**Tamil Nadu**									
1992–93	51.4	70.6	61.8	80.4	94.8	93.0	54.7	82.8	73.0
1998–99	64.4	83.4	78.2	70.0	97.2	95.0	63.9	89.4	84.0
2005–06	78.2	93.4	90.0	95.5	97.5	97.3	83.0	95.4	93.3

The data also indicates considerable rural-urban divide in the use of PNC in India and selected states during 1992–2006. On average, the use of PNC among rural mothers remained lower than their urban counterparts in India. The use of PNC among rural mothers in India increased by 8 percentage points (from 13% in 1992–1993 to 21% in 2005–2006), while it improved by 19 percentage points (33% in 1992–1993 to 52% in 2005–2006) among urban mothers during 1992–2006. Furthermore, the use of PNC remained significantly lower among poor mothers than among their nonpoor counterparts cutting across the rural-urban divide in India during 1992–2006. The use of PNC among rural-poor mothers remained unchanged at an abysmally low level (6% in 1992–1993, 5% in 1998–1999 & 6% in 2005–2006), while it improved marginally by 2 percentage points (8% in 1992–1993, 6% in 1998–1999 & 10% in 2005–2006) among urban-poor mothers in India during 1992–2006. On the other hand, the use of PNC among rural-nonpoor mothers increased by 9 percentage points (from 18% in 1992–1993 to 27% in 2005–2006) as compared to 18 percentage points (from 35% in 1992–1993 to 53% in 2005–2006) among urban-nonpoor mothers in India during 1992–2006.

**Figure 2:**
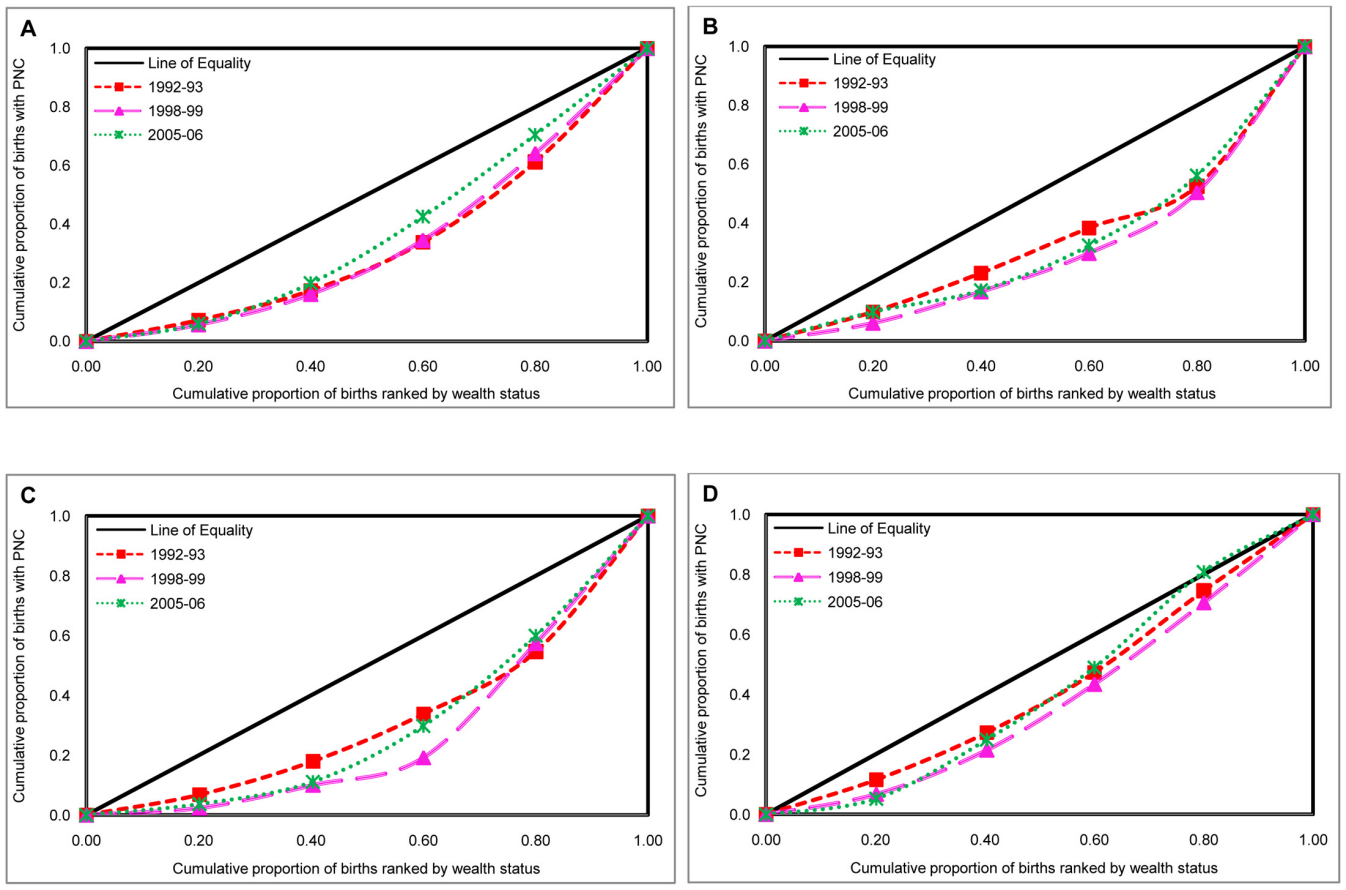
Concentration curves showing inequalities in prenatal care (PNC) by economic status of population across states, India, 1992–2006. **A [INDIA]**. **B [UTTAR PRADESH]**. **C [MAHARASHTRA]**. **D [TAMILNADU]**. X axis = Cumulative proportion of births ranked by wealth status. Y axis = Cumulative proportion of births with prenatal care (PNC). Red square = Concentration curve for 1992–93. Pink triangle = Concentration curve for 1998–99. Green cross = Concentration curve for 2005–06.

We also examined trends in economic inequalities in the use of PNC, measured by concentration indices (CI) and concentration curves (CC), according to the place of residence in India and selected states during 1992–2006 (**Table 3** **&** **Figure 2**). Findings indicate substantially large, consistent and pro-rich inequalities (CI: 0.39, 0.42, 0.35 during 1992–1993, 1998–1999 & 2005–2006 respectively) in the use of PNC among mothers in India during 1992–2006. At the state level, the economic inequalities remained substantially higher in Uttar Pradesh (CI: 0.53, 0.65 & 0.54), followed by Maharashtra (CI: 0.33, 0.33 & 0.20) and least in case of Tamil Nadu (CI: 0.23, 0.19 & 0.11) during 1992–1993, 1998–1999 & 2005–2006 respectively. We further account for the rural-urban differences in economic inequalities in the use of PNC among mothers in India during 1992–2006. Result suggests that economic inequalities in the use of PNC remained precipitously high among rural mothers (CI: 0.35, 0.39 & 0.37) compared to their urban counterparts (CI: 0.25, 0.23 & 0.18) in India during 1992–2006. Among study states, the economic inequality remained high in rural mothers compared to their urban counterparts during 1992–2006. The magnitude of economic inequality remained significantly higher in Uttar Pradesh followed by Maharashtra and was least in the case of Tamil Nadu among both rural and urban mothers during 1992–2006.

**Table 3:** Trends in economic inequalities in prenatal care across selected states, India, 1992–2006.

Indicators	Rural		Urban		Total	
PNC	CI	(SE)	CI	(SE)	CI	(SE)
**India**						
1992–93	0.35	0.009	0.25	0.007	0.39	0.006
1998–99	0.39	0.007	0.23	0.006	0.42	0.005
2005–06	0.37	0.007	0.18	0.005	0.35	0.005
**Uttar Pradesh**						
1992–93	0.33	0.05	0.48	0.031	0.53	0.033
1998–99	0.51	0.055	0.45	0.036	0.65	0.030
2005–06	0.44	0.046	0.40	0.024	0.54	0.021
**Maharashtra**						
1992–93	0.28	0.042	0.31	0.031	0.33	0.024
1998–99	0.41	0.036	0.32	0.017	0.33	0.017
2005–06	0.26	0.028	0.16	0.013	0.2	0.013
**Tamil Nadu**						
1992–93	0.22	0.029	0.17	0.026	0.23	0.020
1998–99	0.17	0.024	0.13	0.017	0.19	0.015
2005–06	0.11	0.019	0.08	0.013	0.11	0.011

### Trends, Differentials and Economic Inequalities in Skilled Birth Attendance (SBA)

The utilization of skilled birth attendance (SBA) among mothers in India, on average, increased by 13 percentage points (from 36.2% in 1992–1993 to 49.5% in 2005–2006) during 1992–2006 (**Table 2**). However, this positive change may be largely attributed to the significant improvement in the uptake of SBA among non-poor mothers (11 percentage points-from 46.4% to 57.8% during 1992–2006) relative to their poor counterparts (2 percentage points-from 17.1% to 18.9% during 1992–2006) during 1992–2006. The use of SBA also varied drastically across Indian states during the study period. On average, the use of SBA varied from the highest in Tamil Nadu (73%, 84%, 93% in 1992–1993, 1998–1999, 2005–2006 respectively) to the lowest in Uttar Pradesh (18%, 23%, 29% in 1992–1993, 1998–1999, 2005–2006 respectively). Importantly, across all the selected Indian states, the use of SBA remained considerably lower among poor mothers relative to their nonpoor counterparts during 1992–2006. For instance, the use of SBA among poor mothers increased by 28 percentage points in Tamil Nadu (from 55% to 83% during 1992–93 to 2005–2006), 12 percentage points in Maharashtra (from 28% to 40% during 1992–1993 to 2005–2006) and 5 percentage points in Uttar Pradesh (from 8% to 13% during 1992–1993 to 2005–2006). On the other hand, among the nonpoor mothers, the use of SBA increased by 13 percentage points in Tamil Nadu (from 82.8% to 95.4% during 1992–2006), 12 percentage points in Maharashtra (from 70% to 82% during 1992–2006), and 10 percentage points in Uttar Pradesh (from 24% to 34% during 1992–2006) during the study period.

Evidence brings out stark rural-urban disparities in the utilization of SBA in India and selected states during 1992–2006. The use of SBA remained significantly lower among rural mothers than among their urban counterparts during the study period. The use of SBA among rural mothers in India registered an increase of 13 percentage points (from 27% to 40% during 1992–2006), while it increased by only 8 percentage points (from 70% to 78% during 1992–2006) among urban Indian mothers. Importantly, the use of SBA remained disappointingly lower among poor mothers across rural-urban spectrum than among nonpoor mothers in India during 1992–2006. For instance, the use of SBA among rural-poor mothers increased marginally by 2 percentage points (from 16% to 18% during 1992–2006), while it surprisingly declined by 5 percentage points (from 36% to 31% during 1992–2006) among urban-poor mothers in India. On the contrary, the use of SBA among rural-nonpoor mothers increased by 14 percentage points (35% to 49% during 1992–2006), compared to 8 percentage points (70% to 78% during 1992–2006) among urban-nonpoor mothers in India during the study period.

**Figure 3:**
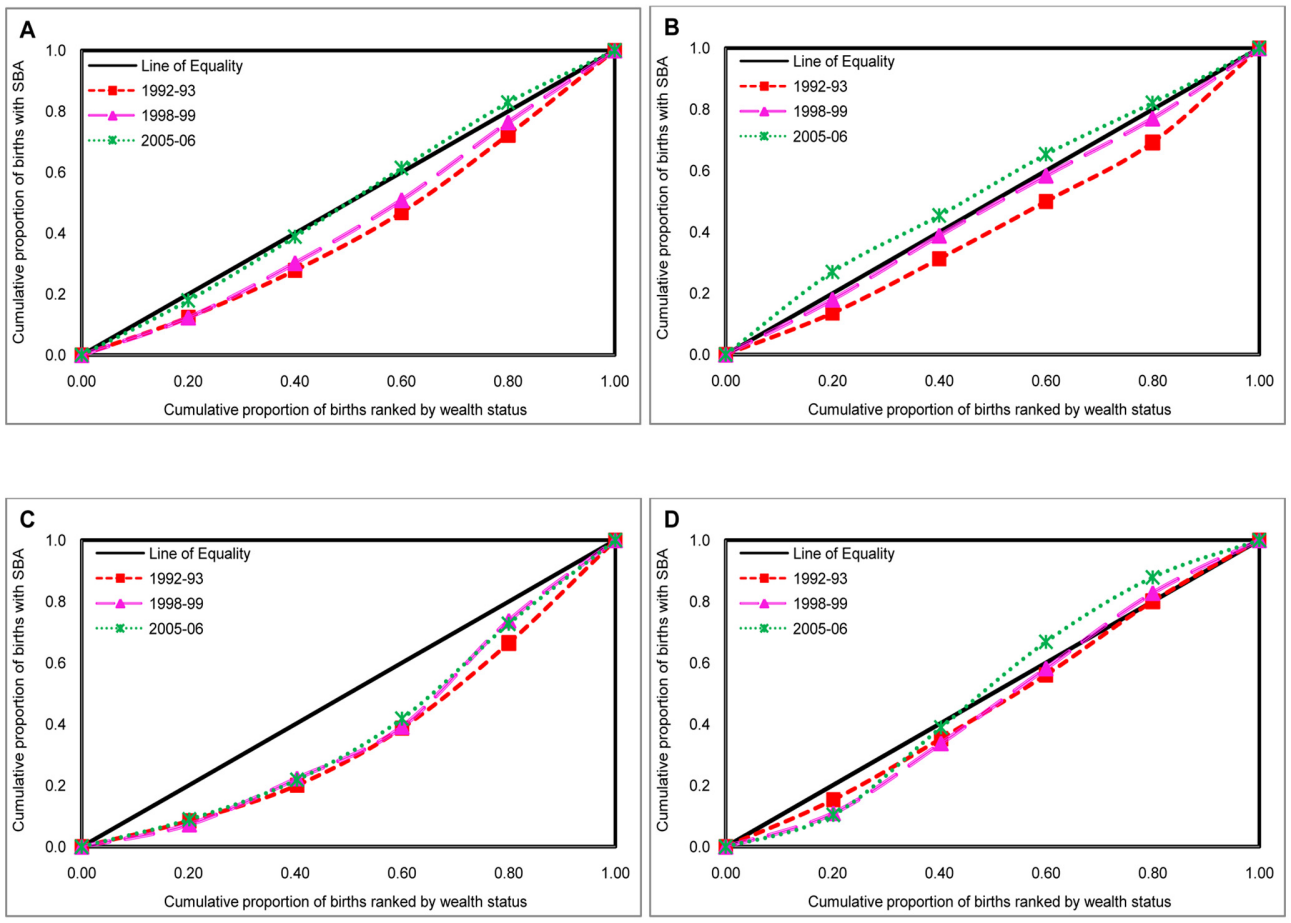
Concentration curves showing inequalities in skilled birth attendance (SBA) by economic status of population across states, India, 1992–2006. **A [INDIA]**. **B [UTTAR PRADESH]**. **C [MAHARASHTRA]**. **D [TAMILNADU]**. X axis = Cumulative proportion of births ranked by wealth status. Y axis = Cumulative proportion of births with prenatal care (PNC). Red square = Concentration curve for 1992–93. Pink triangle = Concentration curve for 1998–99. Green cross = Concentration curve for 2005–06.

In order to measure the degree of economic inequalities in the utilization of SBA during 1992–2006, concentration curves and concentration indices were employed according to the place of residence in India and selected states (**Table 4** **&** **Figure 3**). The inequalities in utilization of SBA remained large and pro-rich in India (CI: 0.39, 0.45, 0.35 during 1992–1993, 1998–1999 & 2005–2006 respectively) during the study period. At the state level, economic inequalities in the utilization of SBA remained considerably higher in Uttar Pradesh (CI: 0.53, 0.65 & 0.54 during 1992–1993, 1998–1999 & 2005–2006 respectively), followed by Maharashtra (CI: 0.33, 0.33 & 0.20 during 1992–1993, 1998–1999 & 2005–2006 respectively), and least in case of Tamil Nadu (CI: 0.23, 0.19 & 0.11 during 1992–1993, 1998–1999 & 2005–2006 respectively). Furthermore, the economic inequalities in the use of SBA remained substantially larger among rural mothers (CI: 0.35, 0.39 & 0.37) than among their urban counterparts (CI: 0.25, 0.23 & 0.18) in India during 1992–2006. Among the study states in general, the economic inequality remained higher among rural mothers relative to urban mothers during 1992–2006. The magnitude of economic inequalities in SBA remained highest in Uttar Pradesh, followed by Maharashtra, and least in the case of Tamil Nadu among both rural and urban mothers during 1992–2006.

**Table 4:** Trends in economic inequalities in skilled birth attendance across selected states, India, 1992–2006.

Indicators	Rural		Urban		Total	
SBA	CI	(SE)	CI	(SE)	CI	(SE)
**India**						
1992–93	0.26	0.005	0.14	0.004	0.31	0.004
1998–99	0.27	0.005	0.12	0.004	0.30	0.003
2005–06	0.25	0.004	0.12	0.004	0.27	0.003
**Uttar Pradesh**						
1992–93	0.24	0.021	0.26	0.018	0.38	0.015
1998–99	0.24	0.024	0.22	0.024	0.34	0.018
2005–06	0.24	0.017	0.26	0.018	0.32	0.013
**Maharashtra**						
1992–93	0.20	0.022	0.10	0.012	0.25	0.013
1998–99	0.27	0.018	0.06	0.009	0.25	0.011
2005–06	0.17	0.014	0.07	0.008	0.17	0.009
**Tamil Nadu**						
1992–93	0.12	0.015	0.04	0.008	0.14	0.01
1998–99	0.08	0.010	0.03	0.008	0.08	0.007
2005–06	0.05	0.008	0.01	0.005	0.03	0.005

### Source of Health Care Providers and Skilled Birth Attendance

We investigate the trends, patterns and changes over time towards the role of health care providers in meeting the demand for skilled birth attendance among mothers by economic status in India and selected states. We have categorised the context of skilled birth attendance into four broad groups: (i) unskilled deliveries at home; (ii) skilled deliveries at home; (iii) deliveries at public health facilities constitutes of the following-delivery at government or municipal hospital; government dispensary; urban health centre (UHC)/urban health post (UHP)/urban family welfare centre (UFWC)/community health centre (CHC)/rural hospital/primary health centre (PHC)/sub centre (SC)/other public health facility; (iv) deliveries at private health facilities constitutes of the following- NGO/Trust hospital/Clinic; Private hospital/maternity home/clinic; other private health facility.

**Figure 4:**
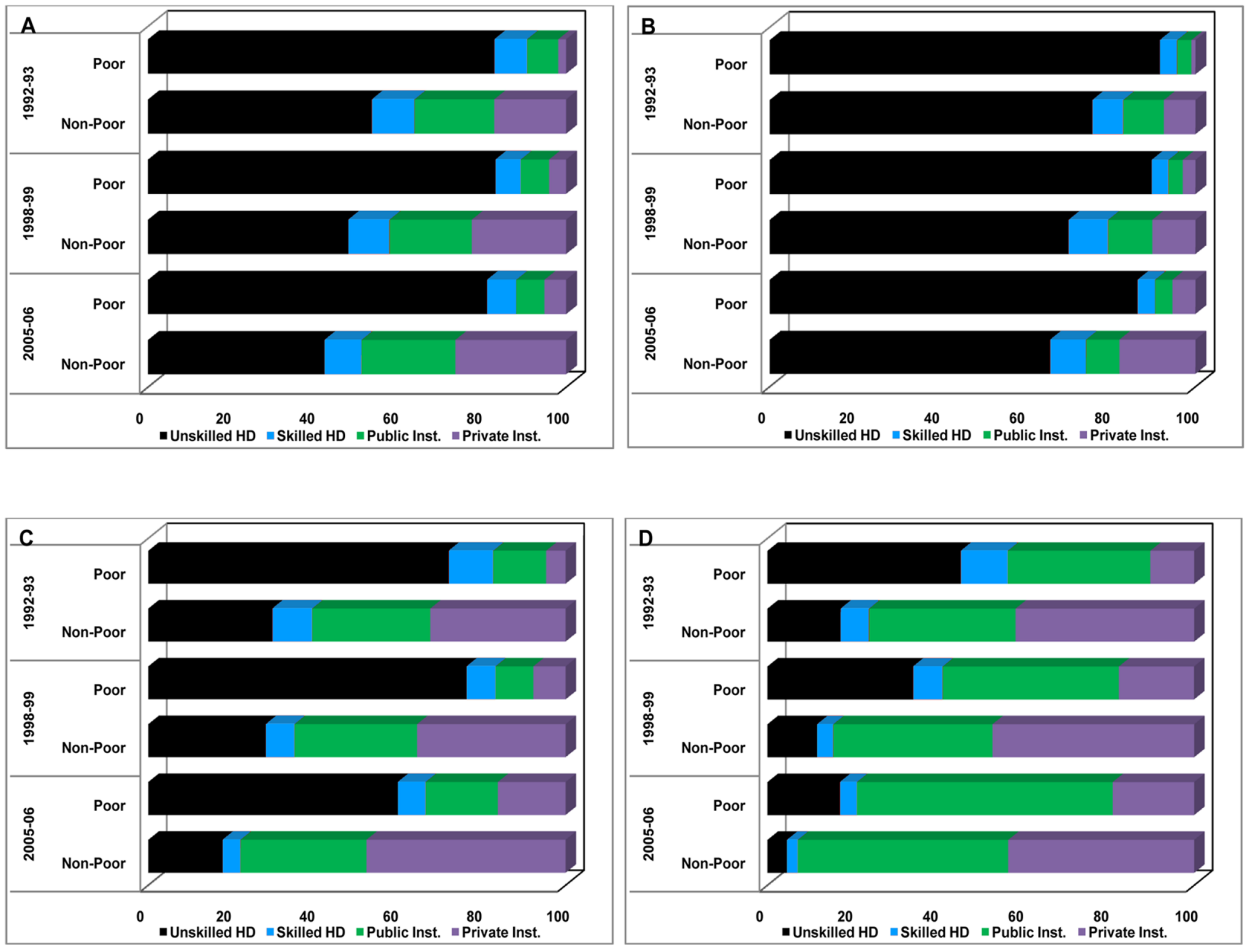
Percent distribution of births delivered by source of providers among poor and non-poor mothers across states, India, 1992–2006. X axis = Economic status [poor vs.non-poor] by survey year [1992–93; 1998–99; 2005–06]. Y axis = Type/place of birth attendance (in percent). Black bar = Unskilled delivery at home. Red bar = Skilled delivery at home. Green bar = Delivery at public health facilities. Purple bar = Delivery at private health facilities.

We present skilled birth attendance by source of health care providers in India and selected states according to economic status and place of residence during 1992–2006 in **Table 5**. Results indicate that, on average, majority of the births in India were at home without the assistance of any skilled medical professional, cutting across economic status, place of residence, and time (**Figure 4**). Less than one-fifth of the deliveries in India were conducted at public health facilities, whereas, about 22% of births were delivered at private health facilities, and around 8% of births were conducted at home with any medical assistance in India during 2005–2006. In general, during the period 1992–2006, the use of private health facility for SBA became more popular (increased from 11.8% to 22.0%) than the use of public health facility (increased from 15.0% to 19.1%). Importantly, among poor mothers, large proportions of births were delivered at home without any medical assistance (82.9% in 1992–1993 to 81.1% in 2005–2006) as compared to nonpoor mothers (53.6% in 1992–1993 to 42.3% in 2005–2006). The use of public health facility for SBA remained significantly higher among the nonpoor than among poor mothers during 1992–2006. We found a negative change in the use of public health facility for SBA among poor mothers, while the private facility for SBA was increasingly used by the poor in India during 1992–2006.

**Table 5:** Percent of birth assisted by skilled health professionals among poor and non-poor mothers by place of residence across selected states, India, 1992–2006.

Time/Indicator	Unskilled home delivery			Skilled home delivery			Delivery at public health facility			Delivery at private health facility		
	Poor	Non-poor	Total	Poor	Non-poor	Total	Poor	Non-poor	Total	Poor	Non-poor	Total
**India (Total)**												
1992–93	82.9	53.6	63.8	7.8	10.2	9.4	7.5	19.1	15.0	1.9	17.2	11.8
1998–99	83.2	47.9	57.1	6.0	9.9	8.9	6.8	19.7	16.3	4.1	22.5	17.7
2005–06	81.1	42.3	50.5	7.0	8.8	8.4	6.7	22.5	19.1	5.2	26.5	22.0
**Uttar Pradesh (Total)**												
1992–93	91.7	75.8	82.2	4.1	7.2	6.0	3.3	9.6	7.1	1.0	7.4	4.8
1998–99	89.8	70.2	76.3	3.9	9.3	7.6	3.4	10.4	8.2	3.0	10.1	7.9
2005–06	86.4	65.9	71.2	4.1	8.4	7.3	4.1	7.8	6.9	5.4	17.9	14.7
**Maharashtra (Total)**												
1992–93	72.0	29.8	44.8	10.6	9.5	9.9	12.7	28.3	22.7	4.7	32.4	22.5
1998–99	76.3	28.2	40.3	6.9	6.8	6.9	9.1	29.4	24.3	7.7	35.6	28.6
2005–06	59.8	17.8	27.8	6.6	4.3	4.8	17.3	30.2	27.1	16.2	47.7	40.2
**Tamil Nadu (Total)**												
1992–93	45.3	17.2	27.0	11.0	6.6	8.2	33.4	34.3	34.0	10.3	41.9	30.8
1998–99	34.2	11.7	16.0	6.9	3.8	4.4	41.3	37.3	38.0	17.6	47.3	41.6
2005–06	17.0	4.6	6.7	4.0	2.5	2.8	60.0	49.3	51.1	19.1	43.6	39.4
**India (Rural)**												
1992–93	84.0	64.3	73.2	7.7	11.4	9.7	6.4	13.8	10.5	1.9	10.6	6.6
1998–99	83.6	56.9	65.9	6.0	10.6	9.0	6.6	15.7	12.7	3.9	16.8	12.4
2005–06	81.6	51.5	59.8	7.0	10.0	9.2	6.4	18.8	15.4	5.1	19.7	15.7
**Uttar Pradesh (Rural)**												
1992–93	92.0	83.5	87.6	3.8	6.3	5.1	3.1	7.0	5.1	1.1	3.1	2.2
1998–99	90.2	76.9	82.0	3.7	7.6	6.1	3.4	8.7	6.7	2.7	6.9	5.3
2005–06	86.5	72.5	76.9	4.1	7.3	6.3	4.2	7.5	6.4	5.3	12.7	10.4
**Maharashtra (Rural)**												
1992–93	72.5	46.7	61.0	11.4	15.6	13.3	10.8	19.5	14.7	5.3	18.3	11.1
1998–99	78.2	42.9	56.1	6.6	10.8	9.2	7.8	21.5	16.4	7.4	24.8	18.3
2005–06	60.3	30.7	41.8	7.8	4.9	6.0	17.2	24.7	21.9	14.7	39.7	30.3
**Tamil Nadu (Rural)**												
1992–93	48.6	29.4	38.2	11.9	11.1	11.5	29.4	27.8	28.5	10.0	31.8	21.8
1998–99	35.7	16.7	21.8	6.1	5.1	5.3	39.7	32.9	34.7	18.6	45.4	38.1
2005–06	21.8	6.6	10.0	5.5	3.4	3.9	57.3	49.3	51.1	15.5	40.6	35.0
**India (Urban)**												
1992–93	63.9	29.9	32.5	7.7	8.3	8.2	25.4	30.5	30.1	3.0	31.3	29.2
1998–99	63.2	25.1	26.2	9.4	8.3	8.3	15.7	29.6	29.2	11.7	37.1	36.3
2005–06	68.6	22.2	23.5	7.2	6.2	6.3	15.2	30.4	30.0	9.0	41.2	40.3
**Uttar Pradesh (Urban)**												
1992–93	71.9	54.2	55.2	14.5	9.8	10.1	13.6	17.0	16.8	0.0	19.0	17.9
1998–99	75.5	46.0	47.3	4.9	15.7	15.2	6.3	16.5	16.1	13.4	21.9	21.5
2005–06	84.4	47.8	48.8	5.2	11.3	11.2	0.0	8.8	8.5	10.4	32.1	31.5
**Maharashtra (Urban)**												
1992–93	56.4	16.7	19.4	5.1	4.6	4.6	38.6	35.2	35.5	0.0	43.5	40.6
1998–99	69.7	14.4	15.4	9.8	3.0	3.2	0.0	37.4	36.7	20.4	45.2	44.8
2005–06	64.5	9.2	11.6	0.4	3.6	3.5	11.7	34.2	33.2	23.5	53.0	51.8
**Tamil Nadu (Urban)**												
1992–93	19.6	5.2	7.0	5.4	1.8	2.3	66.1	40.5	43.8	8.9	52.5	46.9
1998–99	30.0	2.8	5.0	12.0	1.7	2.5	52.0	43.6	44.3	6.0	51.9	48.2
2005–06	4.5	2.5	2.7	0.0	1.5	1.4	64.1	49.7	51.1	31.4	46.2	44.8

We also note considerable rural-urban disparities in the use of SBA by source of health providers in India during 1992–2006. Most of the births in rural areas were delivered at home without any skilled medical assistance (unskilled delivery at home declined from 73.2% to 59.8% during 1992–2006) compared to those in urban areas (unskilled delivery at home declined from 32.5% to 23.5% during 1992–2006). In general, the use of public health facility for SBA remained limited in rural India (changed from 10.5% to 15.4% during 1992–2006). On the other hand, the use of private health facility for SBA made noteworthy increment in both rural (changed from 6.6% to 15.7% during 1992–2006) and urban India (changed from 29.2% to 40.3% during 1992–2006), with larger prominence in the urban areas of the country. Importantly, the use of public health facility for SBA among poor mothers in rural India remained limited (at 6% during 1992–2006) and remained unchanged over the past 15 years, while it significantly declined among urban poor mothers (from 25.4% to 15.2% during 1992–2006) during study period. The utilization of SBA from private health facility has increased significantly among poor mothers, both in rural (from 2% to 5% during 1992–2006) and urban India (from 3% to 9% during 1992–2006). On the other hand, among nonpoor mothers, the use of SBA from public facility has marginally improved by 3 percentage points (from 19.1% to 22.5% during 1992–2006), while, the role of private facility in catering the SBA significantly improved by 10 percentage points (from 17.2% to 26.5% during 1992–2006) in India. The use of private facility for SBA made striking improvement of 9 percentage points (from 10.6% to 19.7% during 1992–2006) in rural areas and 10 percentage points (from 31.3% to 41.2% during 1992–2006) in urban areas in India during 1992–2006. However, stagnation in the use of public facility for SBA among rural-nonpoor mothers and decline in use of SBA from public facility among urban-nonpoor mothers is a cause for concern.

The inter-state variations in the utilization of SBA by source of providers according to economic status of mothers and area of residence suggest large disparities in the use of SBA among poor and nonpoor mothers across Indian states during 1992–2006. On average, the use of SBA from public health facility ranged from a low of 7% in Uttar Pradesh to a maximum of 51% in Tamil Nadu in 2005–2006. On the other hand, the use of private health facility for SBA varied from 15% in Uttar Pradesh to 40% in Maharashtra in 2005–2006. However, unskilled delivery at home ranged from a low of 7% in Tamil Nadu to maximum of 71% in Uttar Pradesh during 2005–2006. Among poor mothers, increment in the use of public facility for SBA varied from a low of 1% in Uttar Pradesh (from 3.3% to 4.1% during 1992–2006), to 5% (from 12.7% to 17.3% during 1992–2006) in Maharashtra and to a maximum of 27% (from 33.4% to 60.0% during 1992–2006) in Tamil Nadu. However, improvement in the use of private facility for SBA among poor mothers ranged from 4% (increased from 1% to 5.4% during 1992–2006) in Uttar Pradesh, 9% in Tamil Nadu (increased from 10.3% to 19.1% during 1992–2006) and 12% (increased from 4.7% to 16.3% during 1992–2006) in Maharashtra. The rural-urban divide in SBA by source of providers across states in India during 1992–2006 remained extensive. Among rural poor mothers, the use of public health facility varied from 4% in Uttar Pradesh to 57% in Tamil Nadu in 2005–2006, whereas it ranged from 5% in Uttar Pradesh to 16% in Tamil Nadu for use of SBA from private facilities. On the other hand, the use of public health facility for SBA among urban-poor mothers varied from almost nothing in Uttar Pradesh to 64% in Tamil Nadu in 2005–2006, while the use of private facility for SBA varied from 10% in Uttar Pradesh to 31% in Tamil Nadu.

### Determinants of Prenatal Care (PNC)

The results from the bivariate analyses provided evidence of a large economic gradient in the utilization of PNC in India during 1992–2006. In order to test whether these observations hold true after adjusting for salient socioeconomic, demographic and cultural variables on the likelihood of seeking PNC, we fitted a binary logistic regression model considering the dichotomous nature of the dependent variable (used PNC = 1; otherwise = 0). We also tested the interaction effects of economic status (poor vs. nonpoor) and time dummies on the likelihood of seeking PNC in India by pooling the data from the three NFHS rounds.

**Table 6** presents the predicted probabilities of seeking PNC for the most recent birth to mothers by place of residence, adjusted for socioeconomic, demographic and cultural characteristics in India during 1992–2006. Results indicate the statistically significant effect of interaction term on the probability of seeking PNC, suggesting that economic inequalities with respect to the use of PNC have changed over time. Poor mothers were significantly less likely to seek PNC than nonpoor mothers. The probability of seeking PNC among poor mothers changed marginally during 1992–2006 (from 0.042 to 0.046). However, during the same period, the probability of seeking PNC increased significantly from 0.170 to 0.327, an increment of around 16 percentage points. Urban mothers were significantly more likely to use PNC than rural mothers. The percentage change in the probability of seeking PNC among urban poor mothers (1.2%) was more than rural poor mothers (0.3%) in India during 1992–2006. On the other hand, percentage change in the probability of using PNC among the urban nonpoor mother (20.1%) was higher than rural nonpoor mothers (11.5%).

**Table 6:** Predicted probabilities of prenatal care adjusted for socioeconomic & demographic characteristics, India, 1992–2006†.

Covariates	Total	Urban	Rural
**Interaction between economic status & time**			
Poor in 1992–93	0.042	0.046	0.040
Poor in 1998–99	0.037	0.037	0.036
Poor in 2005–06	0.046	0.058	0.043
Non-Poor in 1992–93	0.170	0.211	0.153
Non-Poor in 1998–99	0.226	0.322	0.186
Non-Poor in 2005–06	0.327	0.411	0.268
Change among poor, 1992–2006	0.004	0.012	0.003
Change among non-poor, 1992–2006	0.158	0.201	0.115
**Indian states**			
Rest of India	0.193	0.243	0.171
Uttar Pradesh	0.040	0.068	0.028
Maharashtra	0.309	0.330	0.279
Tamil Nadu	0.559	0.579	0.528

† Note: Adjusted for mother education, father education, mother's age at delivery, parity, religion, caste, residence, pregnancy complication, mass media exposure, age difference to head of household.

The probability of seeking PNC was highest among mothers from Tamil Nadu, followed by Maharashtra, and least in the case of Uttar Pradesh. This clearly demonstrates that the use of PNC has varied significantly between states over time. The results for the other covariates were found in the expected directions. Maternal and paternal education, urban residence, mass-media exposure and any form of pregnancy complications were significantly associated with the use of PNC in India. Low parity mothers, with relatively younger age at delivery (<30 years), belonging to non-scheduled caste/non-scheduled tribe were more likely to seek PNC than their counterparts.

### Determinants of Skilled Birth Attendance (SBA)

In order to understand the adjusted effect of theoretically pertinent factors on the likelihood of seeking SBA, and the context of skilled birth attendance, we first fit a binary logistic regression model taking SBA as a dichotomous variable (sought SBA = 1; 0 otherwise). At the second stage, we run a multinomial logistic regression model among births for which the skilled medical attendance was actually sought, taking the context of birth delivery choice as a polytomous variable (home delivery with medical assistance, delivery at public facility and delivery at private facility). **Table 7** presents the predicted probability for seeking SBA, adjusted for salient socioeconomic, demographic and cultural variables in India during 1992–2006. The predicted probability presented in Table 7 suggests that, among poor mothers, the probability of seeking SBA improved marginally by 2 percentage points (from 0.129 to 0.147) during 1992–2006 in India. On the other hand, the probability of seeking SBA among non-poor mothers improved significantly by 19 percentage points (from 0.452 to 0.637). This again confirms that poor mothers continue to suffer more than their nonpoor counterparts when it comes to utilization of maternal health care in India. Results also suggest that the probability of seeking SBA was significantly higher among urban mothers as compared to rural mothers. Among poor mothers in India, the probability of seeking SBA was higher among urban than among rural mothers during the study period.

**Table 7:** Predicted probabilities of skilled birth attendance adjusted for socioeconomic & demographic characteristics, India, 1992–2006†.

Covariates	Total	Urban	Rural
**Interaction between economic status & time**			
Poor in 1992–93	0.129	0.257	0.123
Poor in 1998–99	0.141	0.261	0.139
Poor in 2005–06	0.147	0.214	0.146
Non-Poor in 1992–93	0.452	0.612	0.366
Non-Poor in 1998–99	0.568	0.731	0.481
Non-Poor in 2005–06	0.637	0.753	0.534
Change among poor, 1992–2006	0.018	−0.043	0.022
Change among non-poor, 1992–2006	0.185	0.141	0.168
**Indian states**			
Rest of India	0.451	0.61	0.381
Uttar Pradesh	0.189	0.294	0.162
Maharashtra	0.739	0.846	0.577
Tamil Nadu	0.905	0.962	0.848

† Note: Adjusted for mother education, father education, mother's age at delivery, parity, religion, caste, residence, pregnancy complication, mass media exposure, age difference to head of household.

The probability for seeking SBA was largest in Tamil Nadu, followed by Maharashtra, and least in Uttar Pradesh during 1992–2006. The results for other covariates were found in the expected directions. The likelihood of seeking SBA was significantly higher among births to educated mothers and fathers, urban residents, with mass media exposure, used PNC, had any form of pregnancy complications, low parity, relatively younger age at delivery (<30 years), non-Muslim and non-Scheduled Castes/Scheduled Tribes.

**Table 8** presents the predicted probability from multinomial logistic regression for the choice of delivery, adjusted for socioeconomic, demographic and cultural factors in India. Results indicate that the interaction term between economic status and time dummies had significant influence on the birth delivery choice. In 1992–1993, a large proportion of poor mothers opted for delivery at public health facilities, followed by home delivery assisted by medical professionals, and the rest opted for a private health facility. However, the likelihood of seeking birth at home with medical assistance and public facilities has dwindled significantly, as majority of poor mothers shifted their preference towards the private health facility for deliveries during 1992–2006. The percentage change in the probability of having delivery at a private health facility among poor mothers increased by 20 percentage points (from 0.134 to 0.337) compared to only 7 percentage points (from 0.249 to 0.319) among nonpoor mothers. A similar trend was observed in both urban and rural areas, with extra prominence in urban India. Notably, the likelihood of using a public health facility for SBA among poor mothers reduced significantly, as the percentage decline in the predicted probability of using SBA from public facility was 7 percentage points (from 0.489 to 0.421) during 1992–2006. A similar trend was observed among poor mothers in rural & urban areas. On the other hand, among nonpoor mothers, the percent change in predicted probability of using a public health facility for SBA improved marginally by 3 percentage points (from 0.439 to 0.471). The same trend was observed among rural-nonpoor mothers, but it was not the case among urban-nonpoor mothers. During 1992–2006, the probability of delivery at public health facilities was highest in Tamil Nadu, followed by Maharashtra and least in Uttar Pradesh, while a large proportion of deliveries at home with medical attendance were done in Uttar Pradesh, followed by Maharashtra and least in Tamil Nadu cutting across place of residence. The results for other control variables were in the expected direction.

**Table 8:** Predicted probabilities of birth delivery choice adjusted for socioeconomic and demographic characteristics, India, 1992–2006†.

Covariates	Public health facility	Private health facility	Home delivery assisted by medical professional
**Interaction with economic status & time (Total)**			
Poor in 1992–93	0.489	0.134	0.376
Poor in 1998–99	0.426	0.303	0.271
Poor in 2005–06	0.421	0.337	0.242
Non-Poor in 1992–93	0.439	0.249	0.311
Non-Poor in 1998–99	0.468	0.275	0.257
Non-Poor in 2005–06	0.471	0.319	0.210
Change among poor, 1992–2006	−0.068	0.203	−0.134
Change among non-poor, 1992–2006	0.032	0.070	−0.102
**Indian states**			
Rest of India	0.497	0.249	0.253
Uttar Pradesh	0.354	0.352	0.294
Maharashtra	0.505	0.356	0.139
Tamil Nadu	0.562	0.363	0.075
**Interaction with economic status & time (Urban)**			
Poor in 1992–93	0.663	0.183	0.154
Poor in 1998–99	0.423	0.429	0.149
Poor in 2005–06	0.409	0.514	0.077
Non-Poor in 1992–93	0.490	0.364	0.147
Non-Poor in 1998–99	0.523	0.363	0.113
Non-Poor in 2005–06	0.488	0.427	0.086
Change among poor, 1992–2006	−0.254	0.331	−0.077
Change among non-poor, 1992–2006	−0.002	0.063	−0.061
**Indian states**			
Rest of India	0.532	0.351	0.117
Uttar Pradesh	0.275	0.525	0.200
Maharashtra	0.511	0.454	0.034
Tamil Nadu	0.570	0.411	0.019
**Interaction with economic status & time (Rural)**			
Poor in 1992–93	0.414	0.099	0.486
Poor in 1998–99	0.398	0.236	0.367
Poor in 2005–06	0.386	0.265	0.349
Non-Poor in 1992–93	0.397	0.192	0.410
Non-Poor in 1998–99	0.412	0.236	0.352
Non-Poor in 2005–06	0.445	0.260	0.295
Change among poor, 1992–2006	−0.028	0.165	−0.137
Change among non-poor, 1992–2006	0.048	0.067	−0.115
**Indian states**			
Rest of India	0.450	0.200	0.350
Uttar Pradesh	0.397	0.264	0.339
Maharashtra	0.418	0.310	0.272
Tamil Nadu	0.512	0.360	0.128

† Note: Adjusted for mother education, father education, mother's age at delivery, parity, religion, caste, residence, pregnancy complication, prenatal care, mass media exposure, age difference to head of household.

## Discussion

Over the last two decades, multiple flagship programmes were launched by the Government of India, such as Child Survival and Safe Motherhood Programme (CSSM, 1992–1996), Reproductive and Child Health (Phase I, 1997–2004) and Reproductive and Child Health (Phase-II, 2005–2010) in order to make the life of the mother and neonate safer. The RCH-II programme focussed on narrowing the regional variations in the domain of reproductive and child health, and on the provision of assured, equitable and quality health services to the underserved target population. The central government recently launched the National Rural Health Mission (NRHM, 2005–12) mainly to revamp the rural health infrastructure in 18 low performing states. This programme aims at improving the availability, accessibility, affordability, and quality of effective health care services to the rural population, particularly among poor and underserved women and children. Impetus on institutional deliveries and emergency obstetric care is the key strategy of the central government under NRHM to curb the menace of maternal mortality in the country [79]. However, due to limited evidence on the use of maternal health care over time and across space, it is difficult to evaluate the extent to which these programmatic efforts have benefitted the neediest population subgroups, that is, poor mothers living in rural areas. Therefore, this paper has attempted to examine the trends, patterns and predictors of economic inequalities in the utilization of maternal health care in India and selected states, taking illustrative case of prenatal care and skilled birth attendance, using data from three rounds of Indian National Family Health Survey (NFHS) conducted during 1992–2006. In addition, the relative contribution of the skilled birth attendance providers (public facility vs. private) was also investigated.

The findings from the study revealed a sluggish increment in PNC and SBA in India during 1992–2006. However, the increments were mainly noted among the non-poor mothers, and the poor mothers benefitted least from the government sponsored maternal health care services over the past 15 years. The increment in SBA was largely due to improvement in birth attendance at private health care facilities than at the public health care facilities in India. These trends are alarming and also raise critical questions on the role of the supply side factors related to the public health care system in India. Poor physical accessibility, irregular supplies, absence of adequate staff including lady doctors, lack of continuity from single care giver often force the poor people to shift towards private health facilities [80]. Other studies have reported that nearly 25% of people admitted to hospital become poor because of treatment cost from private health facilities [81]. This further deteriorates the financial condition among the economically poor and pushes them into the vicious cycle of poverty [82], [83]. However, the Government of India has recently started an ambitious conditional cash transfer scheme, the ‘*Janani Suraksha Yojana (JSY)*’, a 100% centrally sponsored scheme under the umbrella of NRHM, to promote institutional delivery, particularly among pregnant women above the age of 19, belonging to below poverty line (BPL) families in both rural and urban areas. According to the JSY scheme, after delivery in a government or accredited private health facility, an eligible woman would receive Rs. 600 and Rs. 700 in urban and rural areas, respectively. The cash incentives for ten high focus-states (Uttar Pradesh, Uttaranchal, Bihar, Jharkhand, Madhya Pradesh, Chhattisgarh, Assam, Rajasthan, Orissa, and Jammu & Kashmir) are set at RS. 1000 in urban areas and Rs. 1400 in rural areas. JSY also provides small financial assistance (Rs. 500) for births at home for pregnant women living below the poverty line and for the first two births. A recent evaluation of JSY suggests that the poorest and least educated women did not always have the highest odds of receiving JSY payments. The evaluation further revealed significant effects of JSY on increasing PNC and in-facility births [84]. However, findings emphasize the need for targeting poor women.

On the other hand, the only true exception to the above phenomenon was Tamil Nadu, where majority of SBA were conducted at public health facilities during 1992–2006, especially among the rural-poor mothers in the state. This largely resulted due to the concerted efforts made by the state government of Tamil Nadu since the early 1990's through various innovative measures like provision of cash incentives worth Rs. 1000/− to pregnant women belonging to below poverty line (BPL) families under the Dr Muthulakshmi Reddy Maternity Assistance Scheme for institutional delivery. A payment of Rs. 50/− per woman was made to Village Health Nurses (VHNs)/Auxiliary Nursing Midwifes (ANMs) if five antenatal visits are provided and institutional delivery was conducted by VHN/ANM [83]. It was further strengthened by the various innovative steps taken by the Government of Tamil Nadu, like surveillance and audit of maternal death, continuum of care from community to first-referral level health facility to shorten the three delays, round-the-clock quality emergency obstetric care facility at the first referral units, maternity picnics to promote delivery at primary health centre, and birth companionship programmes [85], [86].

The utilization of PNC and SBA significantly varied by place of residence, and state of residence in India highlighting the rural-urban disparity and inter-state differentials in the use of maternal health care in India during 1992–2006. The largest improvement in the use of maternal care services were recorded in Tamil Nadu, followed by Maharashtra, while the least change was observed in Uttar Pradesh. Importantly, rural poor mothers were significantly at a disadvantageous position relative to their urban non-poor counterparts in the use of maternal health care. We also found large economic inequality in utilization of prenatal care and skilled birth attendance cutting across space and time in India during 1992–2006. However, economic inequality was more pronounced in the use of prenatal care than skilled birth attendance. This might have occurred due to lack of cash incentives to pregnant women as in the case of SBA, and poor quality of antenatal care services coupled with weak public health systems in the resource poor settings that negatively shape the attitude of women against the use of PNC [87]. Furthermore, the inequality in utilization of prenatal care and skilled birth attendance was mainly prominent in the rural areas than in their urban counterparts in India and the selected states during 1992–2006.

The results from multivariate analyses confirmed that the utilization of maternal health care varied significantly with the economic status of mothers in India. Non-poor mothers from Tamil Nadu or Maharashtra, living in urban areas, with above primary education and literate husband, with low parity and some exposure to mass media were more likely to receive prenatal care than their counterparts in Uttar Pradesh. Further the effect of time dummies was significant and positive, suggesting that mothers who had birth during 2005–2006 were more likely to have PNC than who had births in 1992–1993. We also found that economic status, maternal and paternal education, place of residence, prenatal care, pregnancy complications, mass-media exposure and region of residence had significant effect on the likelihood for seeking SBA.

The findings clearly exert alarming-bells for the public health system in India towards meeting the urgent call of the millions of pregnant mothers for comprehensive, prompt and quality maternal health care, in terms of PNC and SBA services across both rural and urban areas, and also between states, particularly for the poor. The study also highlights the need for regulating the role of the private health sector in India, both in rural and urban areas across states, in catering to the basic need of maternal health care, particularly for poor mothers. Overlooking the lethargy, unpreparedness and inefficiency of public health facilities, and unregulated private health facility in India may exacerbate the high risk pregnancy outcomes and economic distress on the household, particularly among the poor [88]. It is the right time for the government to develop practical models of public-private partnership in order to improve the efficiency, effectiveness and equity in the provision of maternal health care services, derived from the learnings of successful stories such ‘Chiranjeevi Yojana’ in Gujarat. There were very few attempts of its kind in India where, the Government of Gujarat collaborated with the Indian Institute of Management in Ahmedabad, the Society for Education, Welfare and Action – Rural (SEWA Rural), and the German Development Organization (GTZ) to develop a pilot programme to provide skilled birth attendants and emergency obstetric care in five districts. The government hired private obstetricians using simple criteria to provide the quality delivery care to poor women in rural areas [89]. Partnership with the private sector to meet national public health goals is one of the key strategies of the National Rural Health Mission launched by the Government of India in 2005.“Janani” is one successful example of such partnership under the NRHM. Janani leverages private sector resources to supplement public sector service delivery in Bihar. Though these experiments have been successful at local levels, the modalities to up-scale them at the state and national levels need to be worked out.

Finally, we report three key take home messages that come out from the analyses. First, the use of prenatal care and skilled birth attendance remains disproportionately lower among mothers in India during 1992–2006 irrespective of the area of residence and state of residence. Second, despite huge efforts on the part of Government of India and various state governments to increase access and coverage of delivery services to the poor, it is clear that the poor (a) do not use SBA and (b) even if they had SBA, they were more likely to use private providers. The use of SBA with private providers may have occurred due to lack of access to public facilities, perceived poor quality of care at public health facilities (lack of staff, number of staff/24 hour services, rude behaviour), and also due to need for emergency treatment. Lastly, women and particularly poor women obviously make rational decisions given their economic circumstances and this is reflected in the higher use of skilled birth attendance relative to prenatal care. In other words, women recognize the inherent riskiness of delivery and choose to spend their limited budgets on this type of health seeking than on prenatal care.

It is high time that the focus of policy and programme managers should shift from improving the ‘*average figures*’ to the ‘*distribution*’ of programme/health care indicators across the sub-groups of population which need them most. It is the right time when government's policies and programmes start targeting poor and deprived women to address the unmet need for maternal health services among this group of women. The successful example of the Tamil Nadu model may be learned and replicated in other states like Uttar Pradesh, where unskilled birth attendance constitutes more than three fourths of all births in the state, and majority of those who sought SBA used private health care institution during 1992–2006. This calls for urgent enquiry into the supply side variables and quality of care component of the public health delivery system. It will not be possible to meet the unmet need among poor and deprived women without expanding the public health system and without improving the quality of physical and human infrastructure. The public health system must also be ready to address emergency during pregnancy and delivery to encourage poor and deprived women to use public health facilities. Finally, addressing the issue of equity in maternal health care, that continues to pose a formidable challenge at present, may hold the key for the achievement of Millennium Development Goals for India in the near future.

## Supporting Information

Appendix S1 Analytical sample size (unweighted), 1992–2006.
(0.04 MB DOC)
