# Supplementary material for: Economic Inequalities in Maternal Health Care: Prenatal Care and Skilled Birth Attendance in India, 1992–2006
Source: PLoS One. 2010 Oct 27;5(10):e13593. doi: 10.1371/journal.pone.0013593 (PMC2965095; doi:10.1371/journal.pone.0013593)
Supplement: Appendix S1 — Analytical sample size (unweighted), 1992–2006. (0.04 MB DOC) [file pone.0013593.s001.doc]

| Analytical sample | Prenatal care^†^ | Skilled birth attendance^¥^ |
| --- | --- | --- |
| **India** |  |  |
| 1992-93 | 30795 | 34977 |
| 1998-99 | 25528 | 28650 |
| 2005-06 | 25972 | 29396 |
| **Uttar Pradesh^*^** |  |  |
| 1992-93 | 4229 | 4797 |
| 1998-99 | 2293 | 2587 |
| 2005-06 | 3392 | 3930 |
| **Maharashtra** |  |  |
| 1992-93 | 1313 | 1526 |
| 1998-99 | 1420 | 1636 |
| 2005-06 | 1615 | 1804 |
| **Tamil Nadu** |  |  |
| 1992-93 | 1089 | 1233 |
| 1998-99 | 1123 | 1275 |
| 2005-06 | 910 | 1026 |
| Note: ^†^ The sample for prenatal care is restricted to the most recent births in last three years preceding the survey; ^¥^ The sample for skilled birth attendance is restricted to most two most recent births in last three years preceding the survey; ^*^ Since the state of Uttar Pradesh was bifurcated into two parts in 2000, the newly formed state of Uttarakhand (formerly part of Uttar Pradesh) have been excluded from the analysis in 1992-93 & 1998-99. | | |
